# Comparison of Radio- and Phototoxicity in Association with an Enhancing Effect of the Photosensitizers Psoralen, Trioxsalen and Ortho-Iodo-Hoechst33258 on FaDu, PC-3, 4T1 and B16-F10 Cells

**DOI:** 10.3390/biomedicines13010073

**Published:** 2024-12-31

**Authors:** Katja Tietze, Florian Brandt, Kerstin Wetzig, Lisa Hübinger, Marc Pretze, Ralph Alexander Bundschuh, Jörg Kotzerke

**Affiliations:** Department of Nuclear Medicine, University Hospital Carl Gustav Carus, Technische Universität Dresden, 01307 Dresden, Germany; florian.brandt@ukdd.de (F.B.); lisa.huebinger@ukdd.de (L.H.); marc.pretze@ukdd.de (M.P.); ralphalexander.bundschuh@ukdd.de (R.A.B.)

**Keywords:** X-rays, UVA/UVC, photosensitizer, ortho-iodo-hoechst33258, psoralen, trioxsalen, survival fraction, γH2A.X-assay (double-strand breaks), ELISA-assay (cyclobutane pyrimidine dimers)

## Abstract

**Background:** Energy delivered at different wavelengths causes different types of damage to DNA. **Methods:** PC-3, FaDu, 4T1 and B16-F10 cells were irradiated with different wavelengths of ultraviolet light (UVA/UVC) and ionizing radiation (X-ray). Furthermore, different photosensitizers (ortho-iodo-Hoechst33258/psoralen/trioxsalen) were tested for their amplifying effect. Survival fraction and damage analysis using the γH2A.X assay (double-strand breaks) and the ELISA assay (cyclobutane pyrimidine dimers) were compared. **Results:** The PC-3 cells were found to be the most sensitive cells to the treatment strategies used. FaDu and PC-3 showed a strong sensitivity to UVA. Analysis of the damage showed that the cell lines exhibited different sensitivities. **Conclusions:** Thus, an enhancing effect of photosensitizers (PS) in combination with UVA could be demonstrated in some cases. However, this is cell- and dose-dependent.

## 1. Introduction

Energy of different wavelengths can cause extensive DNA damage in biological material. Mutagenicity increases with decreasing wavelength. UVA light (315–400 nm) has been identified as a contributing factor in immunosuppression and is a key initiator in the development of melanoma, basal cell carcinoma and squamous cell carcinoma [1,2,3]. It penetrates deeper into the skin, causing photosensitization and forming covalent bonds between the C5 and C6 of the pyrimidine bases [4]. In contrast, UVC (200–280 nm) increases the formation of cyclobutane-pyrimidine dimers (CPD) and (6-4) pyrimidone photoproducts [2,5]. Compared to this, the electromagnetic irradiation of X-rays results in the formation of reactive oxygen species (ROS) and is in the wavelength range from a few nanometers to picometers [6].

The direct intercalation of PS, such as psoralen (pso) or its derivate trioxsalen (tri), can intensify damage in the DNA and achieve higher irradiation precision [7]. Additional UV light causes the formation of photomonoadducts and crosslinks between two pyrimidine bases and inhibits repair and proliferation [8,9]. In addition, it is known that some PS can transfer energy to thymine in its excited triplet state and thus induce the formation of CPD [10]. Reactive oxygen species (ROS), such as superoxide anion and singlet oxygen, are also among the damaging compounds [11]. Other PS, such as ortho-iodo-Hoechst33258 (oIH), intercalate into the small groove of the DNA [12]. There it is converted to phenylHoechst by dehalogenation and forms carbon-centered radicals. These are analogous to uracil radicals and form double-strand breaks (DSB) [13,14,15].

The aim is to determine the radio- and phototoxicity of external irradiation (X-rays) and light (UVA/UVC) on different tumor cell lines. Furthermore, the enhancing effect of pso, tri and oIH in combination with UVA will be investigated. In addition, the formation of DSB (γH2A.X assay) and CPD (ELISA assay) will be examined.

## 2. Materials and Methods

### 2.1. Cell Culture

FaDu (DD) cells (head and neck cancer, human) were grown in Gibco DMEM (life technologies, Thermo Fisher Scientific, Darmstadt, Germany) with GlutaMax (10% FBS, 1% NEAA (100×), 1% HEPES buffer (10 mM)). These epithelial cells are a subline of the ATCC-derived FaDu cell line (HTB-43), modified and kindly provided by the Baumann group [16]. Furthermore, PC-3 cells (prostate cancer, human, ATCC CRL-1435) were cultured in RPMI 1640 (life technologies, Thermo Fisher Scientific, Darmstadt, Germany) (10% FBS, 1% NEAA (100×)). The ATCC (CRL-2539) 4T1 (breast cancer, mouse) cells were grown in RPMI 1640 (life technologies, Thermo Fisher Scientific, Darmstadt, Germany) (10% FBS) and the B16-F10 (melanoma, mouse; ATCC CRL-6475) were cultured in DMEM (Sigma Aldrich, Darmstadt, Germany) (10% FBS, 1% sodium pyruvate). All cells were grown as monolayers at 37 °C under humidified 5% (*v*/*v*) CO_2_ atmosphere. Cells were detached from the T75 cell culture flasks with trypsin, and the cell number and viability were determined on the Casy TTC (OLS OMNI Life Science GmbH & Co. KG, Bremen, Germany).

### 2.2. Characterization of Cells

To determine the doubling time and the nuclear–cytoplasmic ratio, 100,000 cells were seeded in 6-well multititer plates (MTP) 24 h before the experiment. The cells were then detached and the vitality and cell number were determined. For the nuclear–cytoplasmic ratio, cells were prepared using the Nuclei Isolation Kit: Nuclei EZ Prep (NUC-101, Sigma Aldrich, Inc., Saint Louis, MO, USA) and nucleus and cell sizes were determined on the Casy TTC. Doubling times were calculated in duplicate every 24 h using the Casy TTC.

To detect DNA content, DAPI intensities were measured using AKLIDES^®^ Immunology (software version: 0.2.1_b56; Medipan GmbH/GA Generic Assays GmbH, Dahlewitz, Germany). These are automatically calculated for each nucleus during the γH2A.X foci analysis and are proportional to the DNA content [17]. The fluorescence intensity of at least 300 cells was obtained from each experiment with the same conditions and an exposure time of 120 ms.

### 2.3. PS and Irradiation

Exposures were performed with X-rays from 0.5 to 10 Gy, UVC from 0.001 to 0.1 J/cm^2^ and UVA from 0.5 to 5 J/cm^2^. All X-ray exposures were performed as cultured cells in MTP on the Maxishot Y.TU 320-D03 X-ray tube (Yxlon International GmbH, Hamburg, Germany) (200 kV, 20 mA, self-igniting: 3 mm aluminum and beryllium, collimator plate: WBI, dose rate: 1.294 Gy). For the combination of PS and UVA, the PS (tri 1 nM; oIH 5 nM and pso 10 nM) were incubated for 1 h at 37 °C (in the absence of light) and then irradiated with UVA. All PS were purchased from MedChemExpress, Monmouth Junction, NJ, USA and were pre-dissolved as 10 mM in 1 ml DMSO. The UV irradiation box (Opsytec Dr. Gröbel GmbH, Ettlingen, Germany) was used for UVA and UVC irradiation. UV lamps for UVA (8 pieces) were used: Sankyo Denki Blacklight Blue 352 nm, 15 W, Hiratsuka, Japan and for UVC (4 pieces): Philips TUV 15W/G15 T8, Hamburg, Germany. In addition, dose and power density were measured with the UVA and UVC sensor (RM12 sensor; Opsytec Dr. Gröbel GmbH, Ettlingen, Germany).

### 2.4. Colony Formation Assay

For the colony formation assay, 70,000 cells were treated with either X-rays, UVC, UVA or a combination of PS and UVA irradiation. The cells were then detached and counted. Subsequently, cell type and treatment specific cell numbers were seeded and incubated in 6-well MTPs. The growth time was 7 days for 4T1 and B16-F10, 10 days for FaDu, and 13 days for PC-3, respectively. Cells were fixed with 4% formaldehyde for 15 min and then stained with crystal violet (10 g/L) for 30 min. Colonies (at least 30 cells) were counted under a microscope.

### 2.5. Detection of the DSB Using the γH2A.X Assay

The γH2A.X assay was conducted using a 96-well microtiter plate (MTP) in which 20,000 cells were seeded and treated with X-rays, UVC, UVA, or a combination of PS and UVA. Subsequently, the samples were incubated in 4% formaldehyde in DPBS for 15 min. Between each step, four washes with DPBS were performed. Permeabilization was achieved through the addition of cold 0.1% Triton X-100 in DPBS for 15 min, followed by a 30 min incubation in 1% BSA in DPBS. Furthermore, the primary antibody anti-phospho-histone H2A.X (Ser139) antibody (clone: JBW301, 1 mg/mL, Merck Millipore KGaA, Darmstadt, Germany) was used at a dilution of 1:2000 for 1 h at room temperature. Next, incubation was performed with AlexaFluor 488 goat anti-mouse IgG (2 mg/mL, Invitrogen, Life Technologies GmbH, Darmstadt, Germany) at a dilution of 1:1000 in DPBS for 30 min at 37 °C, accompanied by additional counterstaining of cell nuclei with DAPI (AppliChem GmbH, Darmstadt, Germany) at a dilution of 1:100. Finally, one drop of fluorescence mounting medium (Agilent Technologies, Santa Clara, CA, USA) was added to each well and the plates were then dried in the absence of light. DSBs were enumerated using the AKLIDES^®^ Immunology. At least 300 cells were analyzed for each condition.

### 2.6. DNA Extraction and ELISA Assay

To determine the CPDs, 100,000 cells were seeded in 48-well MTPs and than treated with X-rays, UVC or UVA with and without PS. All plates were incubated for 30 min at 37 °C. The cells were detached and added to the lysis buffer. DNA was extracted using the Tissue DNA Preparation—Column Kit (Jena Bioscience GmbH, Jena, Germany) with modifications. In the first incubation step, a rotation of the Thermomixer Compact (Eppendorf SE, Hamburg, Germany) at 1400 rpm and in the second incubation step at 300 rpm was used. Further modification steps included centrifugation of the lysates for 10 min at 13,000× *g*, removing the DNA twice with 50 μL elution buffer and adding of 50 μL 1x TE-Buffer (100×, Sigma Aldrich, Darmstadt, Germany). To determine the DNA content and purity, 100 μL sample was transferred to the 96-well UV-Star microplate and the absorbance was measured at 260 nm and 280 nm.

For subsequent damage assessment, the uncoated 96-costar assay plates were coated with 0.03 mg/mL protamine sulfate (1400 Heparin-Antidot I.E./mL, 10 mg/mL, LEO Pharma GmbH, Neu-Isenburg, Germany) in ddH_2_O 24 h before the experiment and dried completely in the incubator. Then 1 μg/mL DNA (50 μL per well) was added and dried again. For the detection of CPD damage, the ELISA protocol of Cosmo Bio Co., Ltd., Tokyo, Japan was used and modified according to the circumstances. The modifications included the following steps: anti-CPD mouse IgG2aκ (clone: TDM-2, Cosmo Bio Co., Ltd., Tokyo, Japan) 1:500 in DPBS, goat anti-mouse IgG, biotin (0.75 mg/mL, Life Technologies GmbH, Darmstadt, Germany) 1:500 in PBS-T and streptavidin-HRP (1.25 mg/mL, Life Technologies GmbH, Darmstadt, Germany) 1:5000 in DPBS. The absorbance was measured at 492 nm on the Varioskan Flash (Thermo Fisher Scientific, Vantaa, Finland).

### 2.7. Statistics

Survival and DSB detection assays were performed in triplicate on three different days of each experiment. The analysis of CPD damage was performed on three independent experimental days, each with a single determination of the treated cells and extracted DNA. The detection of CPD was performed on three different days, each with a single analysis. After UVC irradiation (0.1 J/cm^2^), ctDNA (Invitrogen, Darmstadt, Germany) was used as a positive and negative control for the ELISA test system.

All results presented the mean of the single experimental days. For the calculation of survival and DSB, the pooled standard deviation was calculated from the individual standard deviations of the experimental days. Significance levels were determined using the established *t*-test (OriginPro2023b, OriginLab Corporation, Northampton/Wellesley Hills, MA, USA), where *p* < 0.05 was considered significant. These results are shown as normalized to the control cells (without treatment). Furthermore, the combination tests of the damage analysis are shown without the individual treatment with UVA.

## 3. Results

### 3.1. Cell and Nuclear Size, Nuclear–Plasma Ratio, Mean DNA Content and the Doubling Time of the Cell Lines

**Figure 1:**
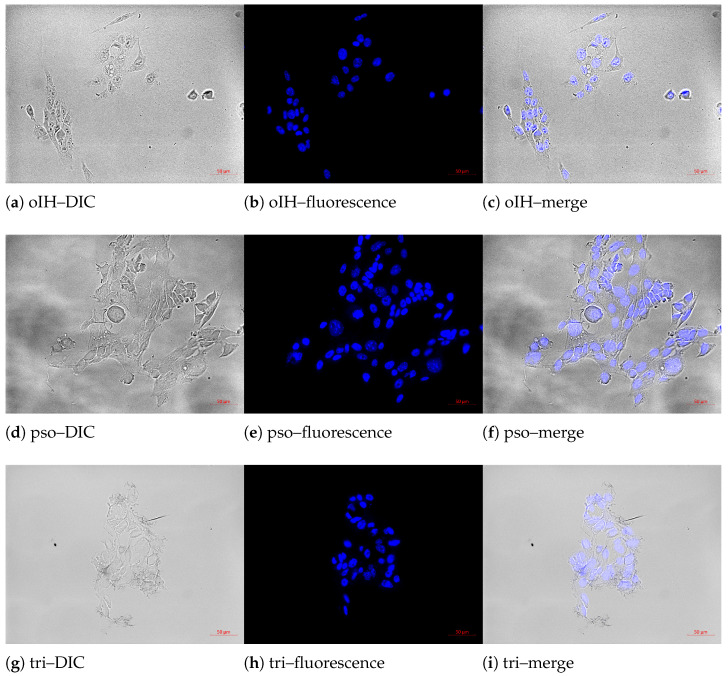
Nuclear staining of 4T1 with oIH, pso and tri. Subsequently, images were captured with the AxioObserver Z.1 fluorescence microscope (Carl Zeiss AG, Oberkochen, Germany; filter set 49; λ_Ex_ = 365 nm, λ_Em_ = 445 nm). Scale: 50 μm. Resolution: 1388 × 1040 pixel, 300 dpi. (**a**,**d**,**g**) DIC with oIH, pso and tri; (**b**,**e**,**h**) fluorescence; (**c**,**f**,**i**) merge images.

Table 1 shows that the human cell lines (FaDu and PC-3) had a larger diameter than the murine cell lines (4T1 and B16-F10). However, there are no differences when comparing the diameters of the nuclei. This resulted in a higher percentage of nuclei in the murine cell lines than in the human cell lines. The average DNA content differed very slightly between the cell lines. The DNA content was lowest in PC-3 and highest in FaDu (factor of 1.03). A comparison of the individual doubling times showed that it was shortest in B16-F10 (17 h) and longest in PC-3 (37 h). This resulted in different colony growth times of 7 days (murine cells), 10 days for FaDu and 13 days for PC-3. Figure 1 illustrates the intercalation of PS into the cell nucleus and binding to DNA using 4T1 as an example.

### 3.2. Determination of the Survival Fraction

**Figure 2:**
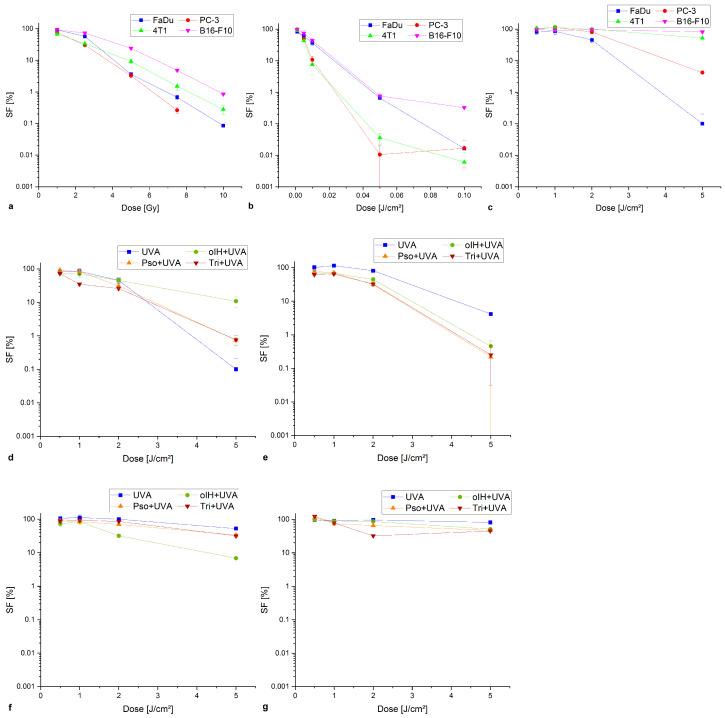
Colony formation assay after treatment with X-rays (**a**), UVC (**b**), UVA (**c**) and with PS + UVA on FaDu (**d**), on PC-3 (**e**), on 4T1 (**f**), on B16-F10 (**g**).

Figure 2 shows colony formation after irradiation with X-rays, UVC, UVA and after PS with UVA.

Figure 2a illustrates the survival fractions following X-ray treatment, wherein the SF exhibited a decline with increasing doses. X-rays had the greatest effect on the PC-3 (7.5 Gy, 0.26% SF). Significant differences were already evident for PC-3 after 1 Gy irradiation (*p* < 0.05) compared to the control. Of the cell lines, PC-3 demonstrated the highest sensitivity to irradiation with 7.5 Gy (0.26% SF) followed by 4T1 (*p* < 0.005 from 2.5 Gy), B16-F10 (*p* < 0.005) from 5 Gy), and FaDu (*p* < 0.05 from 4 Gy) with 0.28%, 0.086% and 0.06% SF at 10 Gy, respectively. A dose–response relationship occurred after treatment with UVC (Figure 2b). After treatment with 0.075 J/cm^2^, B16-F10 demonstrated a low sensitivity (0.32% SF) and a significant difference from 0.0075 J/cm^2^ (*p* < 0.005) while FaDu, 4T1 and PC-3 were sensitive (0.016%, 0.0059%, 0.0017% SF). All three cell lines showed significant differences to the control from 0.005 J/cm^2^ (*p* < 0.05; *p* < 0.005). A single treatment with UVA (Figure 2c) resulted in a dose-dependent decrease in SF in FaDu, PC-3 and 4T1. The highest dose of 5 J/cm^2^ resulted in an SF of 0.1% for FaDu, SF of 4.18% for PC-3 and SF of 52.4% for 4T1. FaDu, PC-3 and 4T1 showed significant differences in increasing order at 2 to 4 J/cm^2^. The B16-F10 did not show a decrease in SF up to 5 J/cm^2^ (81.6%). The addition of PS led to an increased UVA dose–response in PC-3 and 4T1. After treatment with pso and 5 J/cm^2^ UVA, the SF was reduced by a factor of 19.10 compared to single treatment (UVA). Significant differences occurred at a dose of 1–2 J/cm^2^ (*p* < 0.05). Similarly, there was a 16.53-fold reduction in SF after tri and 5 J/cm^2^ UVA and a 9.14-fold reduction after oIH and 5 J/cm^2^. Due to the high effect of UVA as a single treatment, the values are not significant. 4T1 cells showed a comparable increase (7.71-fold) after treatment with oIH and 5 J/cm^2^ (*p* < 0.05). In FaDu, tri and 1 J/cm^2^ UVA (*p* < 0.05), had the most pronounced effect with a 2.43-fold decrease in SF. The combination of pso and 3 J/cm^2^ UVA had the greatest effect at B16-F10 with a 2.04-fold increase (*p* < 0.005).

### 3.3. Damage Analysis on the Example of PC-3 Cells

**Figure 3:**
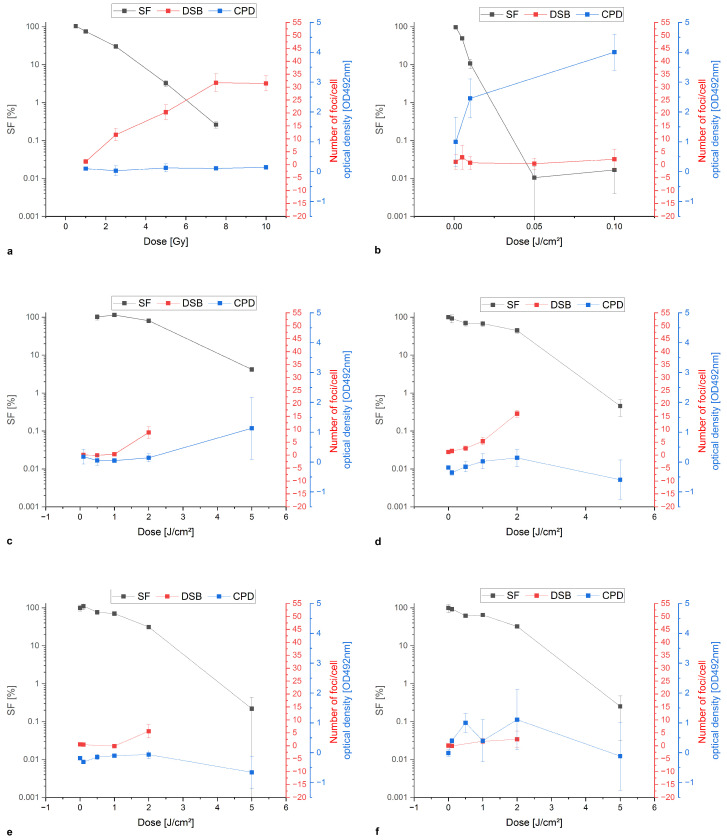
Colony formation assay and damage analysis as a comparison of PC-3 after treatment with X-rays (**a**), UVC (**b**), UVA (**c**), oIH and UVA (**d**), pso and UVA (**e**) and tri and UVA (**f**).

**Figure A1:**
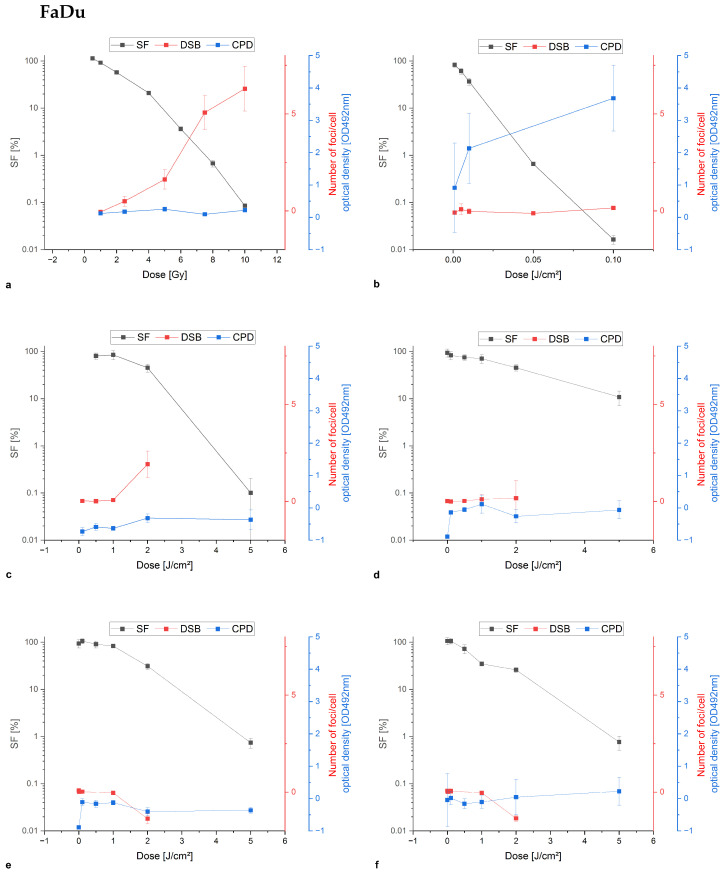
Colony formation assay and damage analysis as a comparison of FaDu after treatment with X-rays (**a**), UVC (**b**), UVA (**c**), oIH and UVA (**d**), pso and UVA (**e**) and tri and UVA (**f**).

**Figure A2:**
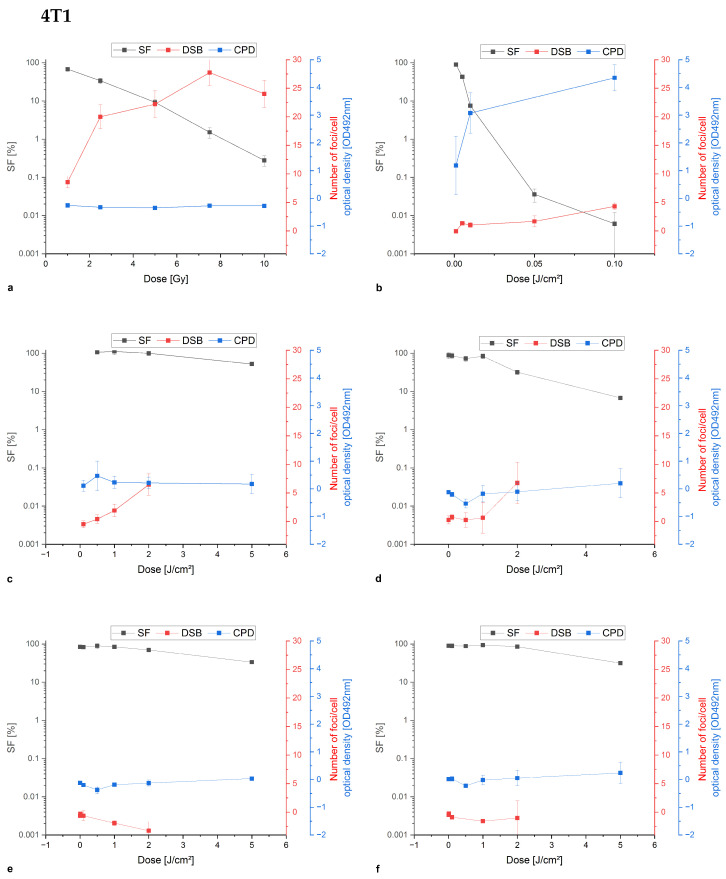
Colony formation assay and damage analysis as a comparison of 4T1 after treatment with X-rays (**a**), UVC (**b**), UVA (**c**), oIH and UVA (**d**), pso and UVA (**e**) and tri and UVA (**f**).

**Figure A3:**
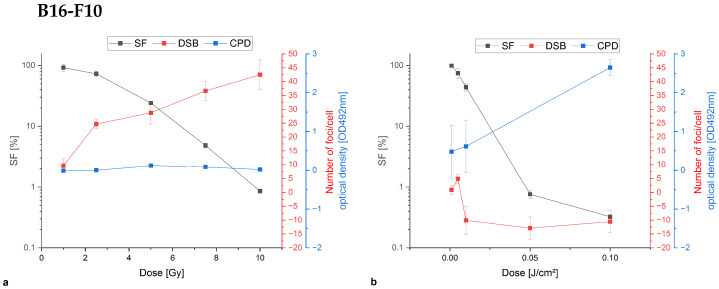

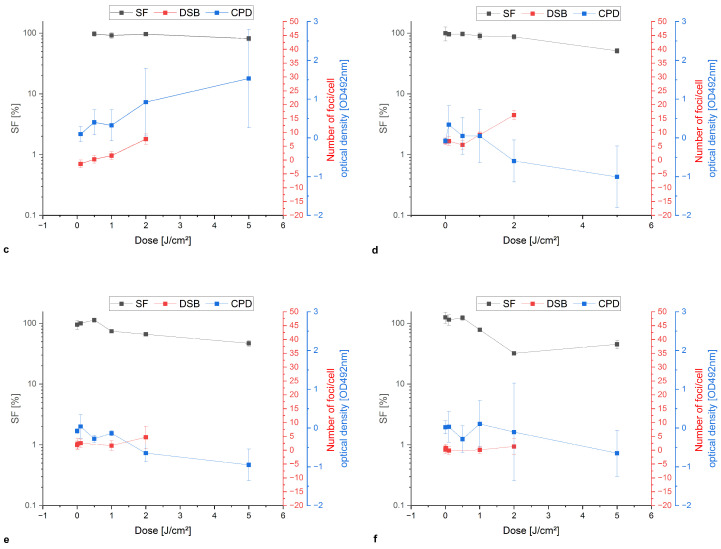
Colony formation assay and damage analysis as a comparison of B16-F10 after treatment with X-rays (**a**), UVC (**b**), UVA (**c**), oIH and UVA (**d**), pso and UVA (**e**) and tri and UVA (**f**).

Figure 3 illustrates the comparison of survival and damage formation of DSB (γH2A.X) and CPD (ELISA test) using PC-3 as an example. The analyses of the cell lines FaDu, 4T1 and B16-F10 can be found in the Appendix A Figure A1, Figure A2 and Figure A3.

After irradiation with X-ray (Figure 3a), no CPD damage was observed up to 10 Gy (OD = 0.07). However, the damage could be quantified by DSB, reaching a maximum of 31 foci per cell at 7.5 Gy (*p* < 0.005), showing an inverse correlation to the SF. In comparison, after treatment with UVC (Figure 3b), a dose-dependent formation of CPD damage can be seen. After a dose of 0.1 J/cm^2^, a maximum of OD = 4.0 is obtained (*p* < 0.005). In contrast, the formation of DSB is very low and reaches a maximum of 2.1 foci per cell (0.1 J/cm^2^). It does not correspond to the decreasing SF. After a single treatment with UVA (Figure 3c), the cells showed the maximum values at the highest dose used. Thus, 5 J/cm^2^ UVA showed CPD damage of OD = 1.1 and 8.8 foci per cell after DSB analysis at 2 J/cm^2^. When oIH was pre-incubated (Figure 3d), the number of DSBs increased by 3.12-fold (1 J/cm^2^) and CPD damage increased by 1.23-fold (2 J/cm^2^). The addition of pso resulted in a 1.51-fold increase in the number of foci at 2 J/cm^2^ (Figure 3e). There was no increase in CPD. In murine cells, there was a slight enhancement of CPD damage when pso and UVA were used. The values are shown in the Appendix. Figure 3f illustrates the increasing number of DSBs of approximately 1.68-fold after the treatment with tri and the additional use of 1 J/cm^2^ UVA and increased the CPD damage about 2.92-fold after 0.5 J/cm^2^ UVA (*p* < 0.05 to control).

## 4. Discussion

The objective of this study was to examine the effect of UV light and external ionizing radiation in vitro to investigate the potential of a combined application to yield a synergistic effect.

X-rays have the most pronounced effect on PC-3, which exhibits a distinctive profile compared to the other cell lines after a dose of 5 Gy. PC-3 cells demonstrate the lowest SF at 7.5 Gy with a value of 0.26%. A similar SF was achieved by FaDu and 4T1 cells only at a dose of 10 Gy. In contrast, the B16-F10 cells are the least radiosensitive and still achieve an SF of 0.86% at 10 Gy. The literature and the present study revealed that treatment with UVC or UVA yielded disparate outcomes when applied to the various cell lines under investigation. The effect of UVC on B16-F10 is markedly less pronounced than on PC-3 [18,19]. It is also noteworthy that UVC irradiation does not provide any indication of the origin of the cells. Both human cells (FaDu) and murine cells (B16-F10) demonstrated reduced sensitivity to UVC, whereas 4T1 (mouse) and PC-3 (human) cells exhibited comparatively heightened sensitivity. The murine cell lines exhibited markedly diminished sensitivity to UVA relative to the human cell lines. Consequently, it can be postulated that UVC irradiation may induce greater DNA damage than UVA. Furthermore, Huang et al. (1998) showed that the SF also decreases from 90% to 18% (0.001 J/cm^2^; 0.004 J/cm^2^) with UVC irradiation. They investigated this in H4E9 cells (clone HT1080) [19]. McMillan et al. (2008) also showed a lower reduction in SF to 85% at 7.5 J/cm^2^ in HaCaT cells [18]. Therefore, a cell type dependency can be assumed.

The SF of the various cell lines after combined treatment with PS and UVA exhibits considerable variation. These differences are largely attributed to the tissue of origin. Additionally, the murine cell lines demonstrate an inherent resistance to the supplementary treatment with various PS and UVA combinations. Enhancement by PS is most evident in PC-3. oIH has a 9.14-fold, pso a 19.10-fold and tri a 16.53-fold enhancement in combination with UVA compared to UVA as a single treatment.Treatment with oIH and UVA also showed a correlation between the concentration of oIH and the amount of light applied. Higher concentrations of oIH require lower doses of UVA to produce similar damage. UVA light induces dehalogenation of the DNA-bound ligand. This leads to the formation of a ligand-specific radical in the small groove of the DNA. This in turn induces DNA lesions initiated by the absence of the deoxyribosyl H atom. As a consequence, the accumulation of DNA lesions leads to cell death. Karagiannis et al. (2006) shows in this context the effect of oIH (among others) and UVA on K562 cells, which show an SF of 40% after 0.1 μM and 0.005 J/cm^2^ [20]. The utilization of pso derivatives also demonstrated an augmentation of the effect and an accompanying reduction in survival. This approach is based on the light quantum yield of pso and tri. As a result, pso achieves a 2-fold higher quantum yield than tri [21]. A potentially higher amount of damage of pso due to the higher quantum yield could be demonstrated especially for human cell lines. However, the difference in the enhancing effects for the murine cell lines are very similar and not significant. The excitation and emission spectra showed a clearly higher intensity of the emission spectrum for tri, suggesting a stronger effect of tri than pso. However, tri also shows a higher intensity in the excitation spectrum. Therefore, a stronger photolysis can be expected for pso and thus a higher quantum yield. The group Jiang et al. (2017) used HEK293T cells to show that the combination of tri and UVA also leads to a reduction in SF. They used a concentration spectrum of 0–328 μM tri and 0.01 J/cm^2^ UVA and achieved an SF of 70% [22].

The analysis of the resulting damage shows some peculiarities, particularly in the PC-3 following X-ray treatment. The outcomes for the initial cells are comparable to those described in the literature. However, there were considerable differences in the residual results. The PC-3 showed a very pronounced repair of the DSB by 94.2%. This value is reported in the literature by approximately 40% of repairing DSB after X-rays. In order to elucidate the elevated repair rate, the status of *TP53* is taken into consideration. A gene that expresses p53 exerts a considerable influence on repair. Given that the repair capacity of the PC-3 utilized in this study is markedly high, it is imperative to ascertain the status of *TP53*. Evidently, this mutation does not occur naturally [23]. In contrast, the FaDu demonstrates a significantly diminished repair capacity (56%) when comparing the initial and residual foci. These were modified by the Baumann group to exhibit a splicing mutation and a single allele of the *TP53* gene. In the absence of this gene and the corresponding p53 protein, or in the presence of a low level of expression, repair is reduced [24].

In contrast, the cells showed a different increase in γH2A.X foci for the initial and residual time points after UVC treatment. Experiments in the literature have shown that a mutation in the *ERCC1* gene is decisive for the initiation of nucleotide excision repair (NER) on CHO-9- and CHO-43-3B-cells. After exposure to 0.002 J/cm^2^ UVC and detection of the kinetics of γH2A.X foci formation [25], other research groups found that there was a steady increase up to 24 h [26]. This indicates the potential of influence of the cell cycle status. The frequency of γH2A.X foci formation is contingent upon the phase of the cell cycle. For instance, cells predominantly exhibit γH2A.X foci during the S phase, which is characterized by DNA replication. It is known that UVC, similar to other genotoxic agents, blocks DNA replication immediately after exposure by inhibiting the start of replication and, at high doses, DNA chain elongation. There is increasing evidence that blocking DNA replication leads to the formation of γH2A.X foci at the sites of blocked replication forks. Therefore, this damage must be repaired before replication starts [25]. The results presented here demonstrate that, at the initial time point, the formation of foci is almost non-existent for all cells. The notable increase over time is particularly evident in PC-3 cells. There is a pronounced downregulation of multiple genes that are involved in both the initiation of repair mechanisms and proliferation. It is shown that the *ERCC1* gene is downregulated after treatment. The results demonstrate that light treatment exerts a discernible impact on cellular processes, influencing the expression of genes and regulating key biological functions such as repair, proliferation and apoptosis [26]. As this involves a different wavelength, a verification by gene analysis should be performed. Nevertheless, the residual curves indicate that the regulation of gene expression differs between the cell lines under investigation. Furthermore, the timing of the assay may also be a significant factor. If the DSBs are not stained immediately after treatment, phosphorylation of the histones may occur after a short time and influence the results. For example, kinetic studies for 8 h after treatment showed the first peaks in DSB formation, which decreased in number by 24 h [27].

The combination of PS and UVA showed a very clear concentration dependency, especially when using oIH and UVA. In the literature, a higher concentration of PS and a lower dose of UVA are usually used. This leads to altered DSB formation, but also to a different formation of CPD damage. Thus, here, only a few CPD formations were observed after the combined treatment of PS and UVA. Above all, these are not dose-dependent. The PS has to be intercalated into the DNA and activated by UVA [27,28,29,30]. UVA irradiation alone shows a dose-dependent formation of CPD damage at high dose points. These occur mainly as TT-dimers. CT and TC dimers, as would have been the result of UVB, cannot be detected [1]. Only UVC irradiation showed the expected CPD damage. This has already been described several times in the literature and corresponds to the results presented here [31].

## 5. Conclusions

In this work, SF after single treatments with X-rays, UVC and UVA were compared to the treatment with PS and UVA. There was a clear difference between the various cell lines depending on their origin. Human cells were particularly sensitive to UVA radiation. The murine cells showed almost no sensitivity to light in the area studied. All cells showed similar sensitivities to the short-wavelength radiation of UVC and the radiotoxicity of X-ray treatment. No species dependence could be determined.

Analysis of the damage showed that the cell lines exhibited different sensitivities. For example, human cells showed a considerable increase in DSBs 24 h after UVC irradiation. The ELISA assay demonstrated the efficacy of the test system in detecting CPD damage induced by UVC irradiation. However, the combination of PS and UVA did not exhibit a dose-dependent increase in damage. Furthermore, it indicates that the system may require further optimization to detect such effects.

Thus, an enhancing effect of PS in combination with UVA could be demonstrated in some cases. However, this is cell- and dose-dependent. A potential chemotoxic effect could be excluded in the concentration range used for colony formation assay. In case of the most sensitive cells (PC-3), the combination of PS and UVA showed a clear increase in damage. For the FaDu cells, an enhancement of PS in combination with UVA was only detectable to a limited extent due to the sensitivity to the single toxin UVA. The murine cells also showed a slight increase in damage. An exception was 4T1 in combination with oIH and UVA. Here, a comparatively high amplification occurred. However, these enhancing effects could not be transferred to the damage analysis. Therefore, it can be assumed that even though there is DSB-caused damage, this is not the main reason for the reduction of the SF. The analysis of CPD damage revealed that it was not the primary factor in the reduction of SF. Therefore, other damage events should be assumed, which must be investigated in further experiments.

Limitations occurred primarily with the PS, which are sometimes very sensitive to light. Furthermore, the UVA light only penetrates the tissue to a small extent (<1 cm). This means that many types of cancer can hardly be reached when used in humans. Further work could focus on the use of PS that are less sensitive to light. This could also have the advantage of using light in the near-infrared range [32]. In the case of the PS used here, the use of radioactivity could be a solution. This could be bound via a peptide-receptor ligand. Light in the UV range should be emitted during decay (Cherenkov light). This could activate the PS and increase the damage. The Maiti et al., 2023 group has already investigated this [33].

The use of oIH in humans poses a considerable challenge. The chemotoxicity test on the cells used showed a clearly higher toxicity than with the two other PS.

Single treatment with oIH showed a noticeable reduction in SF already at 50 nM (FaDu: 50%; PC-3: 80%). In contrast, the individual treatment with tri and pso only achieved comparable SF when using 50 μM. However, tri is already being used on humans. This is performed as part of PUVA therapy (psoralen in combination with UVA) for psoriasis, vitiligo and other skin diseases [34,35].

## Figures and Tables

**Table 1:** Cell and nuclear size, nuclear–plasma ratio, mean DNA content and doubling times of the cell lines.

Cell Lines	Sizes of Cells	Nuclear Size	Nuclear–Plasma Ratio	DNA Content	Doubling Time
	[μm]	[μm]	[x% nuclei]	[AU]	[h]
FaDu	14.75	8.22	55.7	193.3	35
PC-3	16.25	8.83	54.3	187.2	37
4T1	12.58	8.72	69.3	184.1	22
B16-F10	10.83	7.90	72.9	184.0	17

## Data Availability

The data presented in this study are available on request from the corresponding authors.

## Abbreviations

CPD	cyclobutane-pyrimidine dimer
DSB	double-strand break
MTP	multititer plate
NER	nucleotide excision repair
oIH	ortho-iodo-Hoechst33258
PS	photosensitizer
pso	psoralen
ROS	reactive oxygen species
SF	survival fraction
tri	trioxsalen
UV	ultraviolet light
X-rays	external ionizing radiation
